# *Marsupenaeus japonicus* HSP90’s Function Under Low Temperature Stress

**DOI:** 10.3390/biology14080966

**Published:** 2025-08-01

**Authors:** Xueqiong Bian, Xianyun Ren, Shaoting Jia, Tian Gao, Junxia Wang, Jiajia Wang, Ping Liu, Jian Li, Jitao Li

**Affiliations:** 1 State Key Laboratory of Mariculture Biobreeding and Sustainable Goods, Yellow Sea Fisheries Research Institute, Chinese Academy of Fishery Sciences, Qingdao 266071, China; bxq20123@163.com (X.B.); renxianyun301@163.com (X.R.); jiast@ysfri.ac.cn (S.J.); linyigao@163.com (T.G.); wangjj@ysfri.ac.cn (J.W.); liuping@ysfri.ac.cn (P.L.); lijian@ysfri.ac.cn (J.L.); 2 Laboratory for Marine Fisheries Science and Food Production Processes, Qingdao Marine Science and Technology Center, Qingdao 266237, China; 3 College of Fisheries and Life Science, Shanghai Ocean University, Shanghai 201306, China; 4 College of Fisheries and Life Science, Dalian Ocean University, Dalian 116023, China; 5 Marine Science Research Center, Rizhao Ocean and Fishery Research Institute, Rizhao 276800, China; rzdxsyz@163.com

**Keywords:** *Marsupenaeus japonicus*, *MjHSP90*, cold stress, RNAi, apoptosis

## Abstract

Molecular chaperones, especially heat shock proteins (HSPs) have vital functions in cells’ responses to stress. Here, we cloned and sequenced the complete complementary DNA encoding HSP90 (*MjHSP90*) from the shrimp *Marsupenaeus japonicus*. The *MjHSP90* cDNA comprised 3162 bp, including a 2172 bp coding region encoding a 724 amino acid-protein (predicted molecular mass = 83.12 kDa). Homology and phylogenetic analyses showed that MjHSP90 was highly conserved and most homologous to *Litopenaeus vannamei* HSP90. *MjHSP90* is expressed in all tested tissues, with high expression in gill tissue and the hepatopancreas. Cold stress significantly upregulated *MjHSP90* expression in the gill and hepatopancreas (*p* < 0.05). Following RNA interference knockdown of *MjHSP90*, the cold stress-related death rate of the shrimp increased significantly, accompanied by significantly upregulated expression of apoptosis-related genes *Mjcaspase-3* and *Mjbcl-2* (*p* < 0.05) and an increase in the number of apoptotic cells. The results indicated that *MjHSP90* might play a pivotal role in the shrimp’s immune response to cold stress.

## Simple Summary

The increasing frequency and intensity of low-temperature events in aquatic environments have emerged as a critical constraint on sustainable shrimp aquaculture, often leading to substantial economic losses due to reduced growth, impaired physiological functions, and elevated mortality rates in cultured populations. In this study, we investigated the expression pattern of HSP90 under low temperature stress. Moreover, the alterations in the expression levels of genes associated with the mitochondrial apoptosis pathway after RNA interference (RNAi)-mediated MjHSP90 silencing were analyzed to confirm its role in low temperature stress. We demonstrated that heat shock protein 90 plays an important role in the low temperature tolerance of *M. japonicus*, which enhances the body’s low temperature tolerance by reducing the apoptosis induced by low temperature. This finding laid a theoretical foundation for the breeding of a new low temperature tolerant variety of *M. japonicus* and analysis of the cold resistance mechanism of crustacea.

## 1. Introduction

Organisms are critically affected by environmental factors, especially the temperature of water. On the one hand, it can directly affect the growth, feeding, and development of biological life activities, and on the other hand, it can affect the pH, dissolved oxygen, and other environmental factors, which then have an impact on organisms [1,2,3]. Therefore, when organisms are confronted with a sudden change in temperature, they react via a complex set of behavioral, neurological, and physiological responses. These responses are coordinated to restore the homeostatic state and promote the organism’s survival.

The heat shock response (HSR) plays a critical role in the process of temperature stress [4,5,6]. Heat shock proteins (HSPs) are the principal effector molecules of the HSR. Based on their relative molecular mass, HSPs can be classified into five main families: HSP100, HSP90, HSP70, HSP60, and small molecule heat shock proteins (sHSPs) [7]. In response to temperature stress, HSPs function as molecular chaperones, facilitating the maintenance of protein homeostasis through the repair or removal of damaged proteins [8,9].

HSP90, an evolutionarily conserved member of the HSP family, functions as an ATP-dependent molecular chaperone. This structurally dynamic dimer plays a crucial role in facilitating protein folding, modulating signaling pathways, and maintaining interactions with key cellular components, including hormone receptors and regulatory kinases [10,11,12]. Moreover, Hsp90 is among the most abundant cytosolic proteins in eukaryotes, constituting 1–2% of total soluble proteins under non-stress conditions. This high abundance underscores its essential role in cellular homeostasis and function [13,14]. Previous evidence has shown that HSP90 plays an important role in body cold resistance, for example, when HSP90 was knocked down in *Artemia franciscana*, the body’s cold tolerance was significantly reduced [15]. However, HSP90 has been relatively less studied in crustaceans with regard to cold resistance.

The shrimp *Marsupenaeus japonicus* is an economically important crustacean that occurs from southeast Asia and Japan to east Africa and the Red Sea (Bate, 1888). *M. japonicus* are mainly obtained through fishing and farming. *M. japonicus* is farmed in China, Japan, Indonesia, and the Republic of Korea, with China having the highest production, at 45,968 tons, in 2023 [16]. *M. japonicus* is a subtropical farmed shrimp. The optimal thermal range for *M. japonicus* growth is 25–30 °C, within which metabolic activity peaks, feeding efficiency is maximized, and disease incidence remains low. It has been shown to be intolerant to low temperatures, which cause apoptosis and tissue damage and impair development and survival rates [17]. A low temperature environment seriously restricts the development of the shrimp breeding industry. Furthermore, low temperature stress induces HSP90 expression [18,19]; however, the mechanisms underpinning the roles of HSP90 in response to cold stress are unclear.

The Rapid Amplification of cDNA Ends (RACE) technique facilitates the acquisition of full-length cDNAs by specifically amplifying unknown 5’ or 3’ terminal sequences from genes with partially characterized cDNA sequences. Building upon our previous achievement of generating a chromosome-level genome assembly for the *M. japonicus*, we identified a partial sequence of the *HSP90* gene. To extend this finding, the RACE approach was employed to clone the full-length HSP90 cDNA, discussed here. This complete cDNA sequence is anticipated to serve as a molecular foundation for predicting the function of the HSP90 protein (through deduction of its intact coding region) and investigating its regulatory mechanisms (including analysis of the cis-acting elements within the terminal untranslated regions). Consequently, the present study aimed to clone and sequence the full-length cDNA of *M. japonicus HSP90*. We also aimed to characterize its expression patterns under cold stress conditions. Moreover, the alterations in the expression levels of genes associated with the mitochondrial apoptosis pathway after RNA interference (RNAi)-mediated *MjHSP90* silencing were analyzed to confirm its role in low temperature stress. The results partially revealed the role of *MjHSP90* in *M. japonicus* and will contribute to determining the mechanisms of resistance to cold in crustaceans.

## 2. Materials and Methods

### 2.1. Experimental Shrimp Rearing

The Nanshan Seafood Market (Qingdao, China) provided the shrimp (mean body weight of 4 ± 0.5 g, mean body length of 7 ± 0.5 cm). Initially, *M. japonicus* were acclimatized under conditions of 3‰ salinity, pH 8.0 ± 0.2, and 28 ± 0.5 °C for 1 week in polyvinyl chloride polymer tanks containing filtered cycling aerated seawater. The shrimp were provided with fresh clam meat once a day for 7 days (*M. japonicus* of this size were used in all experiments in this study after staging).

### 2.2. Cloning of the MjHSP90 cDNA

We extracted total RNA from *M. japonicus* employing a kit provided by TransGen Biotech (Beijing, China) according to the supplier’s guidelines. We employed a Nanodrop 2000 instrument (Thermo Fisher Scientific, Waltham, MA, USA) to detect RNA purity at A260 nm and A280 nm and its integrity was assessed using agarose gel electrophoresis. A cDNA Synthesis Kit provided by Vazyme (Nanjing, China) was utilized to synthesize first strand cDNA, following the supplier’s guidelines. The program operates at 42 °C for 2 min, at 50 °C for 15 min, and 85 °C for 5 s. A partial cDNA sequence for *MjHSP90* was then amplified by PCR using specific primers and then sequenced commercially by Sangon Biotech (Shanghai, China). The qRT-PCR program operates at 95 °C for 3 min, 30 cycles of 95 °C for 30 s, 55 °C for 30 s, and 72 °C for 5 min, then 10 min at 72 °C and 4 °C. We employed Primer Premier v.5.0 software (Premier Biosoft, San Francisco, CA, USA) [20] to design the primers based on verified *M. japonicus* genome sequences. Subsequently, the full-length cDNA was obtained by rapid amplification of cDNA ends (RACE) employing a SMARTer RACE 5’/3’ Kit (Takara, Shiga, Japan). Table S1 shows the primer sequences. The *MjHSP90* amplicons were sequenced by Sangon Biotech following ligation into the pMD18-T vector (Takara). The full length *MjHSP90* cDNA was assembled from the amplicon sequences.

### 2.3. Sequence Characterization and Phylogenetic Tree Construction

ORF Finder was used to predict the *MjHSP90* cDNA open reading frames (ORFs) [21]. The molecular weight (MW) and isoelectric point (pI) of the encoded proteins were predicted using the ProtParam program [22]. The MjHSP90 aa sequence was deduced employing DNAMAN v.6 (Lynnon Biosoft, San Ramon, CA, USA). The protein sequences of HSP90 from model animals, including *Homo sapiens* (XP_011535020.1), *Danio rerio* (NP_571403.2), *Mus musculus* (NP_034610.1), *Drosophila melanogaster* (NP_001261362.1) and common crustaceans, such as *Litopenaeus vannamei* (ADU03767.1), *Fenneropenaeus chinensis* (ABM92446.1), *Penaeus monodon* (ACO83357.1), *Metapenaeus ensis* (ABR66911.1), *Eriocheir sinensis* (ADE60732.1), *Portunus trituberculatus* (ACQ90225.1), and *Procambarus clarkii* (AGB14568.1), were selected for multiple sequence alignment and phylogenetic analysis. Multiple sequence alignment and results visualization were performed using DNAMAN v.6. The complete alignment method was used for multiple sequence alignment, and the gap penalty strategy was adopted for the gap. With the gap opening penalty set to 10 and the gap extension penalty set to 1A, a phylogenetic tree was produced with support from 10,000 bootstrap replicates, utilizing the neighbor-joining (N-J) technique from a sequence alignment produced by clustaIW in MEGA v.11.0. Phylogenetic tree visualization and annotation were performed using MEGA v.11.0.

### 2.4. Cold Stress and Sample Acquisition

*M. japonicus* were cultured in tanks filled with seawater at 28 ± 0.5 °C, 22 ± 0.5 °C, 16 ± 0.5 °C, and 10 ± 0.5 °C. For each group, we set up three parallel groups of 50 shrimp. Samples of the gill and hepatopancreas were collected at 3, 24, and 48 h following cold stress. Nine shrimp were collected from each temperature group. We utilized an Artificial Climate Chamber (Greete Energy Saving Equipment Limited Company, Weifang, China) to maintain and regulate the water temperature.

### 2.5. Analysis of MjHSP90 Tissue Expression and Low-Temperature Expression Pattern

The relative mRNA expression levels of *MjHSP90* in different tissues (muscle, eyestalk, intestine, heart, gill, stomach, hemocytes, and hepatopancreas), and at various times, were assessed employing the quantitative real-time polymerase chain reaction (qRT-PCR). Initially, RNA was extracted from tissues using the Trizol method (TransGen Biotech, Beijing, China) and cDNA was synthesized utilizing HiScript IV RT SuperMix for qPCR (Vazyme, Nanjing, China). Then, relative *MjHSP90* expression was tested on the LightCycler^®^ 480 Detection System (Roche, Basel, Switzerland) employing ChamQ SYBR Color qPCR Master Mix (Vazyme, Nanjing, China). The qPCR reaction contained 20 µL of 2× ChamQ SYBR Color qPCR Master Mix, 4 µL of cDNA, 1 µL of Primer F, 1 µL of Primer R, and 14 µL of RNase-free water. The qPCR conditions were as follows: initial denaturation (95 °C, 30 s); 40 cycles of 95 °C for 10 s and 60 °C for 30 s; and a melting curve analysis (60 °C for 1 min and 95 °C for 5s). Relative gene expression was calculated using method 2^−ΔΔ*CT*^ using β-actin as the reference gene. The qPCR primers are shown in Table S1.

### 2.6. Analysis of the Localization of MjHSP90 in Gill and Hepatopancreas Tissue

#### 2.6.1. Preparation of Frozen Sections

Gill and hepatopancreas tissues were taken from shrimp under normal conditions, placed in 4% paraformaldehyde solution at 4 °C overnight, and dehydrated the following day in a 30% sucrose solution at 4 °C overnight. Dehydrated tissue was embedded in an optimal cutting temperature (OCT) compound and frozen at −80 °C. Then, 10 µm slices were obtained using a frozen slicer (Leica, Wetzlar, Germany), dried overnight at room temperature, and stored at −80 °C.

#### 2.6.2. Synthesis of the RNA Probe

PCR was used to obtain DNA templates for the synthetic *MjHSP90* RNA probes (Table S1). In vitro transcription of the *MjHSP90* RNA probe utilized a Vazyme T7 RNAi Transcription Kit. RNase-free DNase I and RNase were utilized to digest the in vitro transcription products at 37 °C for 1 h. The resultant product was purified using the spin column method (Omega, Cambridge, MA, USA).

#### 2.6.3. Fluorescence In Situ Hybridization (FISH)

Day1: The tissue sections were rinsed thrice using phosphate-buffered saline (PBS) and PBS-Tween 20 (PBST, 10 min each time) and then placed in hybridization solution (50% deionized formamide, 10% dextran sulfate, 2% 50 × Denhardt’s, 20% 20 × SSC) at 80 °C for 5 min and 70 °C for 15 min. Sections spiked with RNA probes were placed in a wet box for overnight hybridization at 68 °C.

Day 2: Sections were washed three times with wash solution (5% 20 × SSC, 50% deionized formamide, 0.1% Tween20) at 70 °C for 30 min each and with TNT (1 M Tris-HCl, 5M NaCl, 20% Tween20) for 10 min each. To block endogenous oxidase activity, the sections were incubated with glycine-HCl (pH 2) for 10 min. The sections were washed three times with TNT for 10 min each. The sections were then incubated in a wet box with blocking solution (TNT with 2% sheep serum) at ambient temperature for 3 h and then with anti-digoxigenin-POD (1:2000, TransGen Biotech, Beijing, China) for overnight hybridization at 4 °C.

Day 3: Sections were washed four times with TNT for 5 min each. Confinement and color development using fluorescein labeling kits (Beyotime, Shanghai, China) were carried out according to the manufacturer’s instructions. The sections were washed four times with TNT for 5 min each, stained using 4′,6-diamidino-2-phenylindole (DAPI), blocked utilizing anti-quencher, sealed with nail varnish, and stored at 4 °C. The results were observed by means of laser confocal microscopy (Leica).

### 2.7. The Impact of MjHSP90 Knockdown on the Expression Levels of Genes Associated with the Mitochondrial Apoptosis Pathway

#### 2.7.1. Double-Stranded RNA (dsRNA) Synthesis

PCR was utilized to obtain DNA templates for synthetic *MjHSP90* double-stranded RNA (dsRNA) (dsMjHSP90) and green fluorescent protein dsRNA (dsGFP) construction, employing the primers presented in Table S1. A T7 RNAi Transcription Kit (Vazyme, Nanjing, China) was employed for in vitro dsRNA transcription, following the manufacturer’s protocols. Thereafter, RNase-free DNase I and RNase were used to process the in vitro transcription products at 37 °C for 60 min. Magnetic beads were used to purify the products, followed by determination of their concentrations.

#### 2.7.2. Detection of dsMjHSP90 Interference Efficiency

Thirty shrimp were injected with dsGFP and thirty with dsMjHSP90, at 1 µg/g of shrimp body weight. We collected gill and hepatopancreas samples at 24, 48, and 72 h after injection. The effects of *MjHSP90* interference were tested using qRT-PCR.

#### 2.7.3. *M. japonicus* Mortality When Treated with Cold Stress After MjHSP90 Knockdown

Shrimp were assigned to four groups (*n* = 80 per group): the dsMjHSP90 injection group reared in water at 10 ± 0.5 °C, the dsGFP injection group reared in water at 10 ± 0.5 °C, the saline injection group reared in water at 10 ± 0.5 °C, and the saline injection group reared in water at 28 ± 0.5 °C. At 1 day after dsRNA injection, cold stress was initiated. Mortality counts were recorded at 3 h, 24 h, 48 h and 72 h post-cold-stress initiation.

#### 2.7.4. Assessment of Gene Expression Associated with the Mitochondrial Apoptosis Pathway Following MjHSP90 Knockdown

Shrimp were assigned to four groups (*n* = 80 per group): the dsMjHSP90 injection group reared in water at 10 ± 0.5 °C, the dsGFP injection group reared in water at 10 ± 0.5 °C, the saline injection group reared in water at 10 ± 0.5 °C, and the saline injection group reared in water at 28 ± 0.5 °C. At 1 day after dsRNA injection, cold stress was initiated. Gill and hepatopancreas tissues were sampled at 3, 24, and 48 h following cold stress. qRT-PCR was utilized to determine the expression levels of genes associated with the mitochondrial apoptosis pathway.

### 2.8. TUNEL Assay Detection of Apoptosis

Gill and hepatopancreas tissues were processed following previously described protocols [23,24]. In brief, following 24 h of incubation in 4% paraformaldehyde, the gill and hepatopancreas samples were embedded in paraffin, sectioned at 5 µm, stained using hematoxylin and eosin, and finally observed with the aid of a microscope. Paraffin-embedded tissue sections were subjected to deparaffinization, digested using proteinase K (Servicebio, Wuhan, China), stained with DAPI, and subjected to a terminal deoxynucleotidyl transferase nick-end-labeling (TUNEL) assay (Servicebio, Wuhan, China) before microscopic examination.

### 2.9. Statistical Considerations

The presented data are mean values calculated using three independent experiments and are presented as the mean ± standard deviation (SD). One-way analysis of variance (ANOVA), utilized to determine the statistical significance between groups, was carried out employing GraphPad Prism v.8.0 software (GraphPad Inc., La Jola, CA, USA). A *p*-value < 0.05 was employed to indicate statistical significance. The line graphs were created employing GraphPad Prism software.

## 3. Results

### 3.1. Analysis of the MjHSP90 Sequence

Figure S1A shows the whole cDNA and proposed protein sequence of MjHSP90. The full-length *MjHSP90* cDNA comprises 3162 bp, encompassing a 5′ untranslated region (UTR) of 104 bp, an ORF of 2172 bp, and a 3′ UTR of 886 bp (GenBank accession number PQ539624). The ORF translates to a predicted protein of 724 amino acids (aa) (predicted pI = 4.8 and MW = 83.12 kDa. Five conserved HSP90 protein family signatures were found in the deduced amino acid sequence of HSP90: NKELISNSSDALDKIR (33–49), LGTIAKSGT (99–108), and IGQFGVGFYSAYLVAD (124–140) were located at the HATPase_C domain; IKLYVRRVFI (352–362) and GVVDSEDLPLNISRE (379–394) were located in the HSP90 domain. We used SMART for domain analysis of HSP90. The results are shown in Figure S1B, which contains the HATPase_C domain and the HSP90 domain.

### 3.2. Multiple Sequence Alignment

The results of the multiple sequence comparison revealed high similarity between MjHSP90 present in other species (Figure S2). The MjHSP90 amino acid sequence was not particularly similar to *Danio rerio* HSP90 (21.49%) but was highly similar to HSP90 protein sequences from other crustaceans, for example, *Penaeus monodon* (97.38%), *Litopenaeus vannamei* (97.38%), *Fenneropenaeus chinensis* (96.71%), and *Metapenaeus ensis* (96.69%).

### 3.3. Phylogenetics

**Figure 1:**
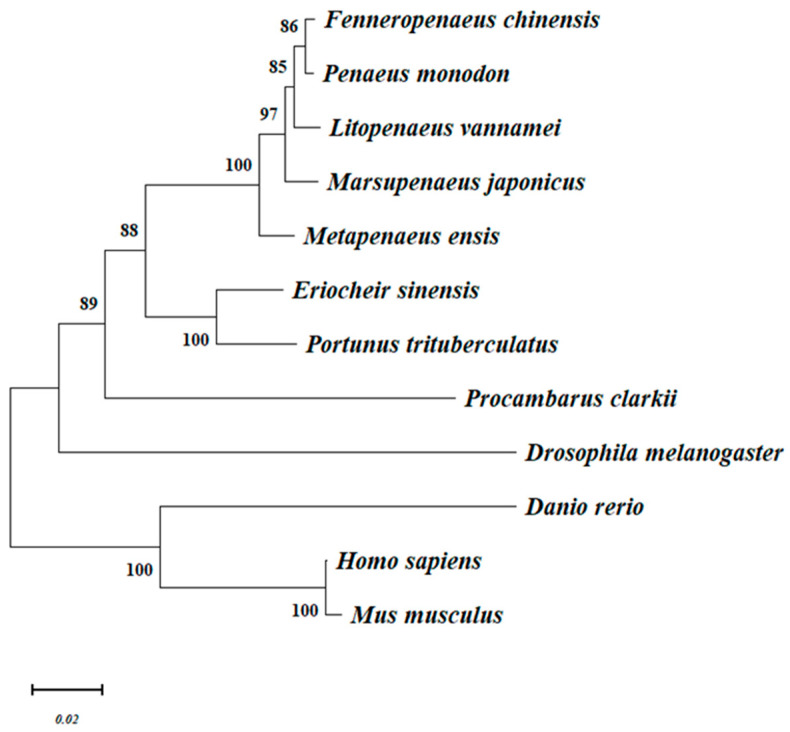
Phylogenetic analysis of MjHSP90. *Danio rerio* (NP_571403.2), *Metapenaeus ensis* (ABR66911.1), *Eriocheir sinensis* (ADE60732.1), *Portunus trituberculatus* (ACQ90225.1), *Procambarus clarkii* (AGB14568.1), *Litopenaeus vannamei* (ADU03767.1), *Fenneropenaeus chinensis* (ABM92446.1), *Penaeus monodon* (ACO83357.1), *Homo sapiens* (XP_011535020.1), *Mus musculus* (NP_034610.1), *Drosophila melanogaster* (NP_001261362.1). Numbers at each branch indicate the percentage of times a node was supported in 1000 boostraps pseudoreplication by neighbor joining.

The phylogenetic tree mainly gathered two major groups: vertebrates and arthropods, in which *Drosophila melanogaster* was separately clustered to form a sister group to the crustaceans. MjHSP90 was located in the crustacean subgroup, which was closer to the Penaeus sequence. Phylogenetics indicated that MjHSP90 was closely related to HSP90s from *L. vannamei*, *P. monodon,* and *F. chinensis*. MjHSP90 also clustered with HSP90s from *M. ensis* (Figure 1). The results were in good agreement with the concepts of traditional taxonomy.

### 3.4. Tissue Distribution of MjHSP90 in M. japonicus

**Figure 2:**
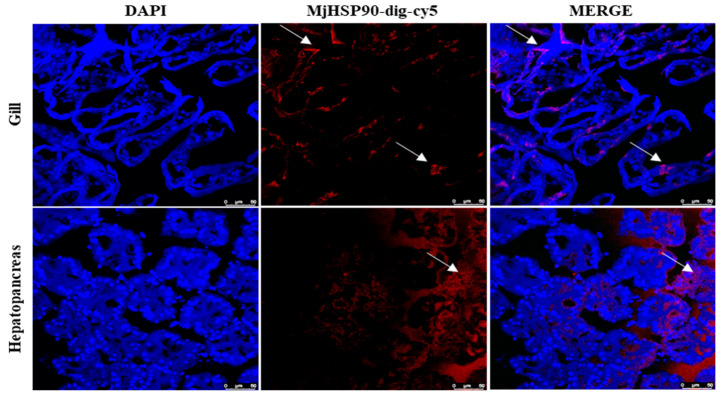
Location of the *MjHSP90* mRNA in *M. japonicus* gill and hepatopancreas tissues. DAPI, 4′,6-diamidino-2-phenylindole; MjHSP90, *M. japonicus* heat shock protein 90. The arrow represents mRNA localization of *HSP90* in gills and hepatopancreas.

*MjHSP90* expression was observed in all tissues tested, showing the highest expression levels in gill tissues, followed by the hepatopancreas, heart, and intestine (Figure S3). The FISH results demonstrated that *MjHSP90* had fluorescent signals in *M. japonicus* gill and hepatopancreas tissue with the presence of a localization marker (Figure 2).

### 3.5. Expression Pattern of MjHSP90 in Cold Stress-Treated Shrimp

**Figure 3:**
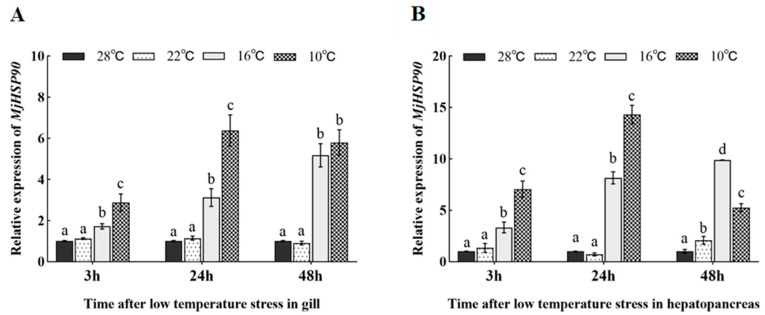
Expression pattern of *MjHSP90* in *M. japonicus* gill (**A**) and hepatopancreas (**B**) samples after cold stress. Bars indicate the mean ± S.D (*n* = 3). Different letters above the bars indicate significant differences between groups at *p* < 0.05, calculated using analysis of variance (ANOVA) (*n* = 3).

Under cold stress, *MjHSP90* expression showed similar trends in *M. japonicus* gill and hepatopancreas tissues: increasing with decreasing temperature (Figure 3). *MjHSP90* expression in the gill and hepatopancreas did not change significantly in the 22 °C group compared with that in the 28 °C control group (Figure 3). *MjHSP90* expression was consistently upregulated in the hepatopancreas and gill tissues when incubated at 16 °C, with a peak at 48 h (Figure 3, *p* < 0.05). *MjHSP90* expression was maintained at a high level during stress at 10 °C in both the gill and the hepatopancreas, showing a tendency to increase and then decrease, with peak expression at 24 h (Figure 3, *p* < 0.05).

### 3.6. The Efficiency of the MjHSP90 dsRNA Interference

**Figure 4:**
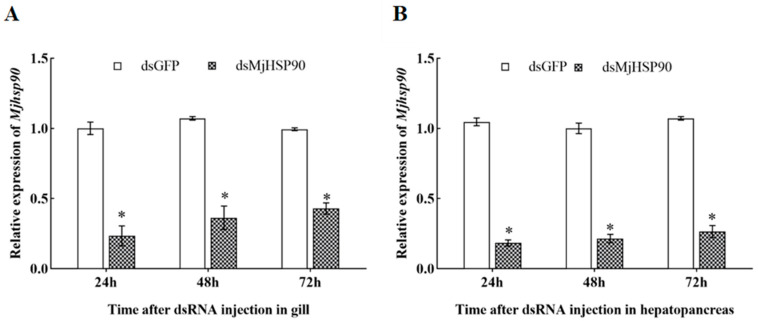
Efficiency of *MjHSP90* dsRNA interference in *M. japonicus* gills (**A**) and hepatopancreas (**B**). Bars indicate the mean ± S.D (*n* = 3). Asterisks (*) above the bars indicate significant differences between groups at *p* < 0.05, calculated using ANOVA (*n* = 3). GFP, green fluorescent protein; dsRNA, double-stranded RNA.

The interference targets were designed, and pre-experiments were conducted to confirm the interference efficiency and duration of action. The gene was successfully knocked down in gill and hepatopancreas tissues, with interference efficiencies of 57.19% and 73.52%, respectively, at 72 h after injection (Figure 4, *p* < 0.05).

### 3.7. M. japonicus Mortality When Treated with Cold Stress After MjHSP90 Knockdown

**Figure 5:**
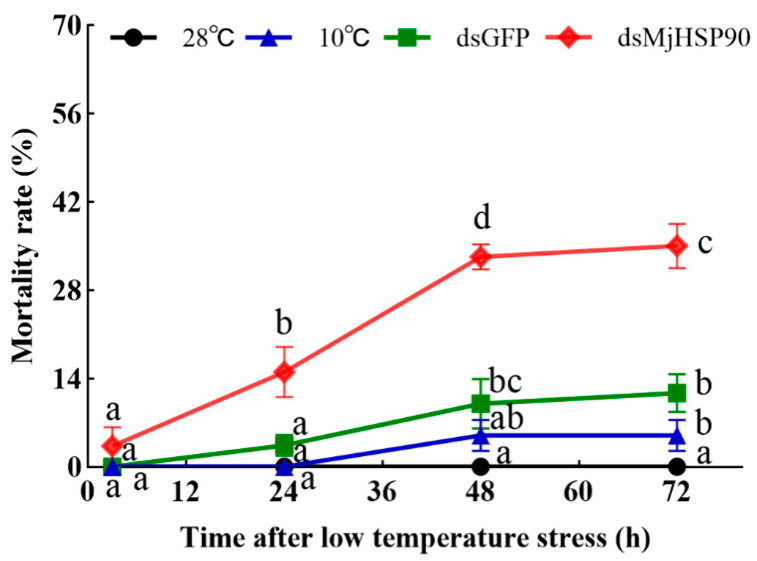
*M. japonicus* mortality under cold stress at different times following *MjHSP90* knockdown. Bars indicate the mean ± S.D (*n* = 3). Different letters above the bars indicate significant differences between groups at *p* < 0.05, calculated using ANOVA (*n* = 3).

As shown in Figure 5, after 72 h of cold stress there was no mortality in the 28 °C group, 5% and 11% in the 10 °C group and dsGFP group, respectively, and 35% in the dsMjHSP90 interference group, which was significantly higher than that observed in the dsGFP group (*p* < 0.05).

### 3.8. Differential Expression of M. japonicus Apoptotic Pathway Genes Under Cold Stress After MjHSP90 Knockdown

**Figure 6:**
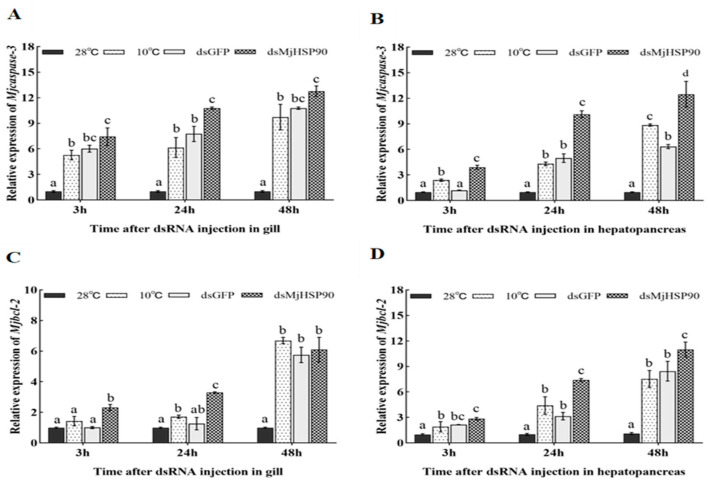
Effect of *MjHSP90* knockdown on the expression of *Mjcaspase-3* and *Mjbcl-2* in *M. japonicus* gills (**A**,**C**) and hepatopancreas (**B**,**D**) under cold stress. Bars indicate the mean ± S.D (*n* = 3). Different letters above the bars indicate significant differences between groups at *p* < 0.05, calculated using ANOVA (*n* = 3).

As shown in Figure 6B, after *MjHSP90* knockdown, we observed a significant increase in *Mjcaspase-3* expression in hepatopancreas tissue relative to that observed in the dsGFP group (*p* < 0.05), whereas significant changes were observed in gill tissues only at 24 h (Figure 6A). After *MjHSP90* knockdown, the expression of *MjBcl-2* was consistently upregulated in the gills and significantly increased compared with that in the dsGFP group at both 3 and 24 h (Figure 6C, *p* < 0.05). Moreover, the expression of *MjBcl-2* was augmented markedly in the hepatopancreas compared with that in the dsGFP group at both 24 and 48 h (Figure 6D, *p* < 0.05).

### 3.9. Cell Apoptosis Following MjHSP90 Knockdown

**Figure 7:**
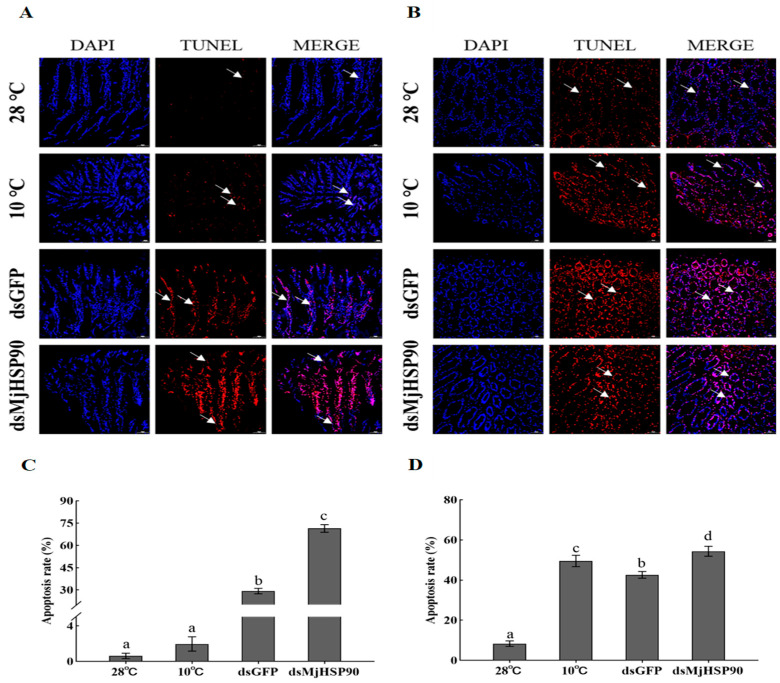
Detection of apoptosis in *M. japonicus* gills (**A**,**C**) and hepatopancreas (**B**,**D**) under cold stress after *MjHSP90* knockdown. Bars indicate the mean ± S.D (*n* = 3). Significant differences between groups at *p* < 0.05 (*n* = 3, ANOVA) are indicated by different letters above the bars. TUNEL, terminal deoxynucleotidyl transferase nick-end-labeling.

The TUNEL assay showed that the least apoptotic cells were observed in the hepatopancreas and gills of the group treated with 28 °C; however, as the temperature was reduced, more apoptosis cells were observed (Figure 7). The apoptosis rate statistics showed that the apoptosis rate in the gills in the lower-temperature groups increased significantly relative to that in the group treated with 28 °C following 24 h under cold stress, such as a 1.96% increase in the group treated with 10 °C, a 29.31% increase in the dsGFP group, and a 71.44% increase in the dsMjHSP90 group (Figure 7C, *p* < 0.05). In the hepatopancreas, the apoptosis rate of the other groups was significantly increased after cold stress for 24 h relative to that in the group treated with 28 °C, including an apoptosis rate of 49.55% in the 10 °C group and 42.63% in the dsGFP group. In the dsMjHSP90 group, the apoptosis rate increased significantly to 54.39% relative to that of the dsGFP group (Figure 7D, *p* < 0.05).

## 4. Discussion

We identified and analyzed the *MjHSP90* cDNA sequence. The *MjHSP90* cDNA and deduced protein sequences are highly homologous to those from *P. monodon*, which has the same status as the traditional classifications. Moreover, the deduced protein sequence contains conserved HSP90 family features and conserved structural domains (Pfam: HSP90). Both the alignment results and phylogenetic tree analysis showed that MjHSP90 had a stronger homology with HSP90 previously identified from crustaceans.

*HSP90* is expressed in different tissues of organisms with a high degree of variability [25,26]. For example, its expression patterns vary under environmental stress and during development [26,27]. The results of the tissue distribution analysis showed that *MjHSP90* was expressed in all tissues tested, hinting that it might play important roles in various vital activities of the organism.

Evidence suggests that multiple factors induce *HSP* expression [28,29,30,31]. Here, the *MjHSP90* gene expression level increased as the temperature decreased, indicating its involvement in the *M. japonicus* cold stress response. Similar findings were obtained in Zebrafish [28], *Salvelinus namaycush* [32], *Triplophysa siluroides* [33], *Phoxinus lagowskii* [34], *Artemia franciscana* [15], *L. vannamei* [18], and *Macrobrachium rosenbergii* [19]. The gill is exposed directly to the water environment, and the hepatopancreas serves as a key organ for detoxification and immunity in crustaceans [35,36]. Therefore, it is important to determine the expression of *MjHSP90* in these tissues. *MjHSP90* expression was significantly higher under 16 °C and 10 °C stress than that in the 28 °C group (*p* < 0.05). After cold stress for 48 h, *MjHSP90* expression in the 10 °C group was not significantly changed or was even lower than that in the 16 °C group. We speculate that this might be due to the earlier massive induction of *MjHSP90* transcription, because at 24 h, *MjHSP90* expression in the group treated with 10 °C was markedly augmented compared with that in the group treated at 16 °C (*p* < 0.05). Together, these findings suggest that *HSP90* has vital functions in aquatic animals’ responses to cold stress and in body homeostasis maintenance.

HSPs are believed to be major proteins used by aquatic animals to respond to environmental stress [17]. Previously, we showed that cold stress augmented apoptosis-associated gene expression, especially *Mjcaspases-3* and *Mjbcl-2*, which induced apoptosis, and increased the expression of HSPs [37]. Consequently, we studied the impact of *MjHSP90* on the mitochondrial apoptotic pathway using *MjHSP90* knockdown after cold stress. Our findings indicated that *MjHSP90* knockdown significantly increased the expression levels of genes associated with apoptosis and exacerbated gill and hepatopancreas cell apoptosis. Similarly, *caspase-3* and *caspase-7* were also significantly increased under heat stress after interference with expression of the *SaHSP90* gene in the *Seriola aureovittata* [38].

Thus, the results of this study showed that cold stress markedly induced *MjHSP90* expression in both the hepatopancreas and gill. Moreover, following *MjHSP90* knockdown, we observed significant increases in *Mjcaspase-3* and *Mjbcl-2* expression in the hepatopancreas and gill in comparison with that in the control group. This suggested that MjHSP90 improves *M. japonicus* cold tolerance by mediating caspase-dependent mitochondrial apoptosis.

## 5. Conclusions

In summary, the *MjHSP90* cDNA was cloned and subjected to bioinformatic analysis. Domain prediction demonstrated that MjHSP90 has conserved domains typical of the HSP90 family. *MjHSP90* is widely expressed in various tissues and is highly expressed in the gill. Low temperature upregulated *MjHSP90* expression in the hepatopancreas and gill. *MjHSP90* knockdown significantly upregulated the expression levels of genes associated with apoptosis and increased apoptotic cell numbers induced under low temperature, eventually resulting in augmented individual mortality. The results suggested that MjHSP90 plays a pivotal role in the immune response of *M. japonicus* to cold stress.

## Data Availability

The sequence data of *MjHSP90* have been stored in the GenBank database with approval number PQ539624.

## Supplementary Materials

The following supporting information can be downloaded at: https://www.mdpi.com/article/10.3390/biology14080966/s1. Figure S1: A: Complete nucleotide and deduced amino acid sequence of *MjHSP90* in *M. japonicus*. Green letters represent HSP90 family signature; Grey letters represent conservative structural domain (Pfam: HSP90); The ATPase domain of MjHSP90 was underlined; The open boxes indicate start codons, stop codons and putative polyadenylation signaling sites. B: Schematic representation of the HSP90 domain; Figure S2: Multiple sequence alignment of the deduced aa sequences of MjHSP90 with other known HSP90 sequences; Figure S3: Tissue distribution of *MjHSP90* in *M. japonicus*. hepatopancreas (Hp), hemocytes (He), gill (G), stomach (S), intestine (I), heart (H), muscle (M) and eyestalk (E). Each bar represents the mean ± S.D (*n* = 3). Significant difference between groups at *p* < 0.05 (*n* = 3, ANOVA) are indicated by different letters above the bars; Table S1: Primers used for cDNA cloning, dsRNA synthesis, qPT-PCR and RNA probe synthesis.
