# Supplementary material for: Marsupenaeus japonicus HSP90’s Function Under Low Temperature Stress
Source: Biology (Basel). 2025 Aug 1;14(8):966. doi: 10.3390/biology14080966 (PMC12383600; doi:10.3390/biology14080966)
Supplement: Supplementary file 1 [file biology-14-00966-s001.zip › Supplementary Figures-revised-2nd.pdf]

# Figure S1

A

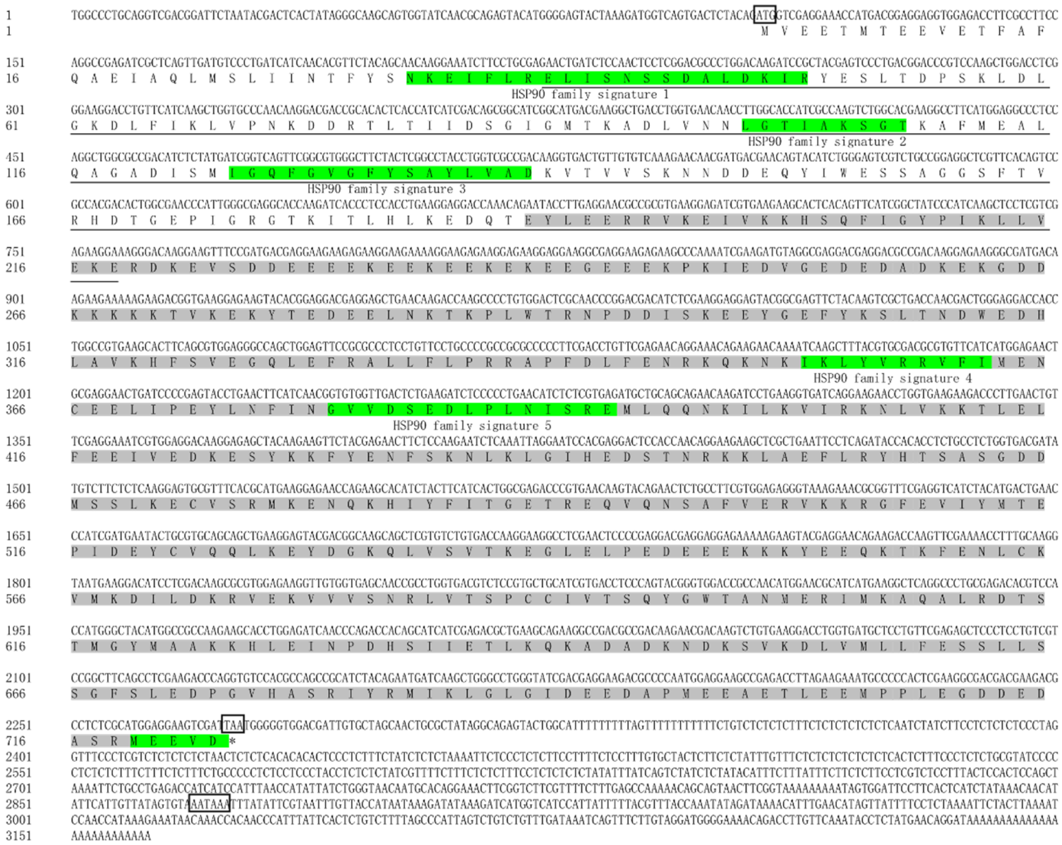

HSP90 family signature 1
HSP90 family signature 2
HSP90 family signature 3
HSP90 family signature 4
HSP90 family signature 5


B

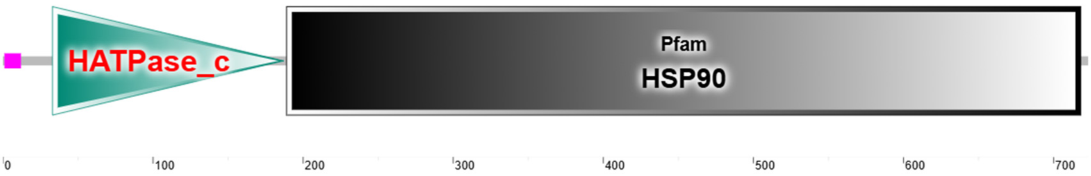

HATPase_c
Pfam
HSP90
0
100
200
300
400
500
600
700

# Figure S2

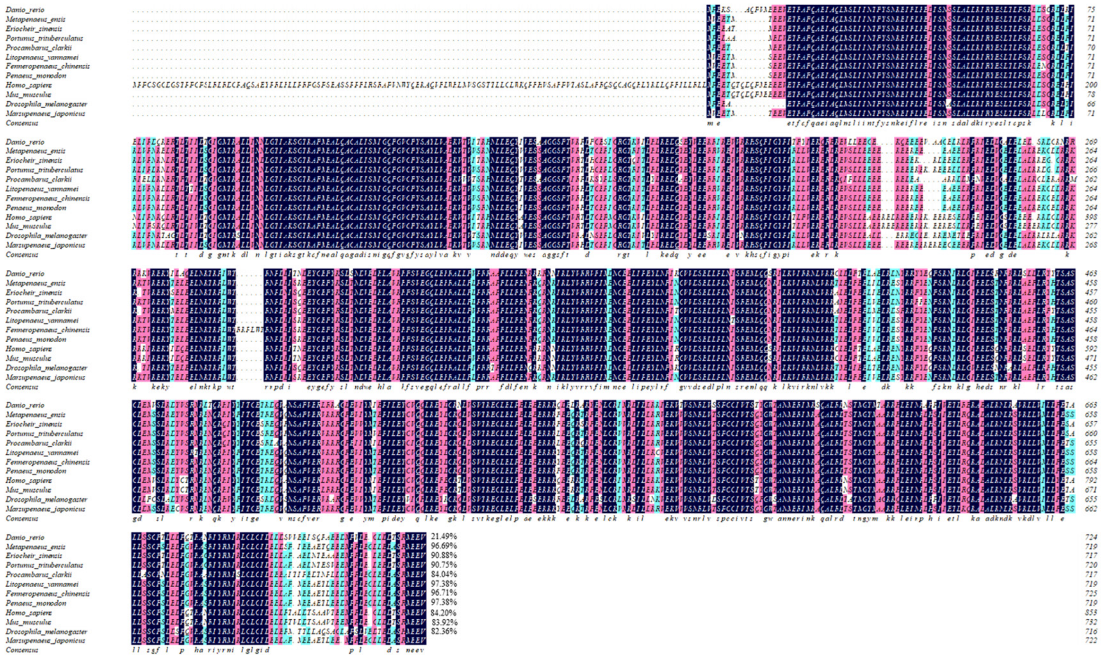
Danio_rerio
Metapenaeus_ensis
Eriocheir_sinensis
Portunus_trituberculatus
Procambarus_clarkii
Litopenaeus_vannamei
Fenneropenaeus_chinensis
Penaeus_monodon
Homo_sapiens
Mus_musculus
Drosophila_melanogaster
Marsupenaeus_japonicus
Consensus

Figure S3

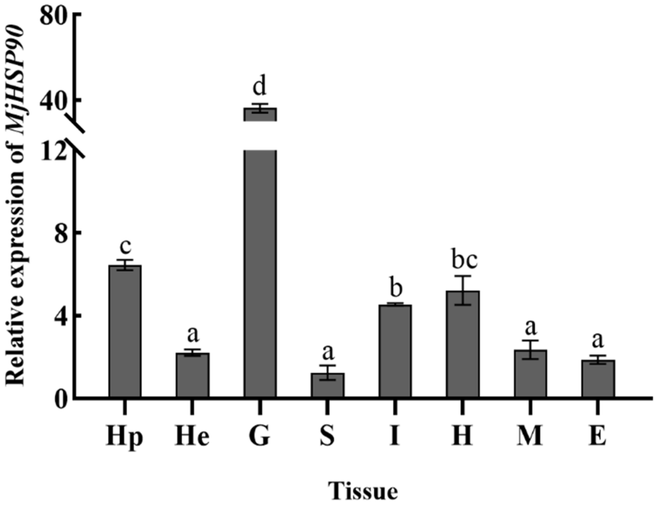
Relative expression of MjHSP90
80
40
12
8
4
0
c
a
d
a
b
bc
a
a
Hp
He
G
S
I
H
M
E
Tissue

**Figure captions**

**Figure S1** A: Complete nucleotide and deduced amino acid sequence of *MjHSP90* in *M.japonicus*. Green letters represent HSP90 family signature; Grey letters represent conservative structural domain (Pfam: HSP90); The ATPase domain of MjHSP90 was underlined; The open boxes indicate start codons, stop codons and putative polyadenylation signaling sites. B: Schematic representation of the HSP90 domain

**Figure S2** Multiple sequence alignment of the deduced aa sequences of MjHSP90 with other known HSP90 sequences.

**Figure S3** Tissue distribution of *MjHSP90* in *M. japonicus*. hepatopancreas (Hp), hemocytes (He), gill (G), stomach (S), intestine (I), heart (H), muscle (M) and eyestalk (E). Each bar represents the mean ± S.D (n = 3). Significant difference between groups at $p < 0.05$ (n = 3, ANOVA) are indicated by different letters above the bars.
