# Supplementary material for: Marsupenaeus japonicus HSP90’s Function Under Low Temperature Stress
Source: Biology (Basel). 2025 Aug 1;14(8):966. doi: 10.3390/biology14080966 (PMC12383600; doi:10.3390/biology14080966)
Supplement: Supplementary file 1 [file biology-14-00966-s001.zip › Supplementary Tables-revised-2nd.pdf]

Table S1. Primers used for cDNA cloning, dsRNA synthesis, qPT-PCR and RNA probe synthesis.

Primer	Sequence (5'-3')	Usage
MjHSP90 F	ATGGTCGAGGAAACCATGACG	sequence verification
MjHSP90 R	TTAATCGACTTCCTCCATGCGAGAG	sequence verification
MjHSP90-5' F1	GATGTCGGCGCCAGCCT	5'-RACE
MjHSP90-5' F2	GGCAGACGACTCCCAGAGT	5'-RACE
MjHSP90-3' R1	GTGGTGAGCAACCGCCTG	3'-RACE
MjHSP90-3' R2	GTGTCCACGCCAGCCG	3'-RACE
UPM (short)	CTAATACGACTCACTATAGGGC	RACE
UPM (long)	CTAATACGACTCACTATAGGGCAAGCA GTGGTATCAACGCAGAGT	RACE
MjHSP90 F	TGGCGAGACCCGTGAACAAGTA	qRT-PCR
MjHSP90 R	ACCAGGCGGTTGCTCACCACAA	qRT-PCR
MjCaspase-3 F	GCTCCGTCGTTGTCGTCGTCTT	qRT-PCR
MjCaspase-3 R	CTTGGGCTTGCCTTTCATTTGC	qRT-PCR
MjBcl-2 F	TCCACGAGACCACATACAAC	qRT-PCR
MjBcl-2 R	CACTTCCTGTGAACGATTGA	qRT-PCR
β-Actin F	TCCACGAGACCACATACAAC	qRT-PCR
β-Actin R	CACTTCCTGAACGATTGA	qRT-PCR
MjHSP90 F	GAGGAAACCATGACGGAGGAGGT	probe
MjHSP90 R	TAATACGACTCACTATAGG GCTTGATGGGATAGCCGATGAACT	probe
dsMjHSP90 F	GATCACTAATACGACTCACTATAGGG CCATCGATGAATACTGCGTG	RNAi
dsMjHSP90 R	GATCACTAATACGACTCACTATAGGG GTCTTCCTCGTCGATACCCA	RNAi
dsGFP F	GATCACTAATACGACTCACTATAGGG ACCCTCGTGACCACCCTGAC	RNAi
dsGFP R	GATCACTAATACGACTCACTATAGGG TCTCGTTGGGGTCTTTGCTC	RNAi
